# Aspartate aminotransferase/alanine aminotransferase ratio was associated with type 2 diabetic peripheral neuropathy in a Chinese population: A cross-sectional study

**DOI:** 10.3389/fendo.2023.1064125

**Published:** 2023-02-27

**Authors:** Pijun Yan, Yuru Wu, Xiaofang Dan, Xian Wu, Qian Tang, Xiping Chen, Yong Xu, Jianhua Zhu, Ying Miao, Qin Wan

**Affiliations:** ^1^ Department of Endocrinology, The Affiliated Hospital of Southwest Medical University, Luzhou, China; ^2^ Metabolic Vascular Disease Key Laboratory of Sichuan Province, Luzhou, China; ^3^ Sichuan Clinical Research Center for Nephropathy, Luzhou, China; ^4^ Cardiovascular and Metabolic Diseases Key Laboratory of Luzhou, Luzhou, China; ^5^ Southwest Medical University, Luzhou, China

**Keywords:** aspartate aminotransferase/alanine aminotransferase ratio, diabetic peripheral neuropathy, vibration perception threshold, type 2 diabetes mellitus, Chinese population

## Abstract

**Objective:**

Despite previous research that focused on aspartate aminotransferase/alanine aminotransferase ratio (AAR) as predictors of type 2 diabetes mellitus (T2DM) and cardiovascular disease, there has been limited research evaluating the association between AAR and diabetic microvascular complications. This study aimed to investigate the association of AAR with diabetic peripheral neuropathy (DPN).

**Methods:**

A total of 1562 hospitalized patients with T2DM were divided into four groups according to AAR quartiles. The relationship between AAR and DPN and related parameters was explored by the Spearman correlation coefficients, multivariable logistic regression analysis, and receiver operating characteristic (ROC) curves.

**Results:**

Patients with higher AAR quartiles had higher levels of vibration perception threshold (VPT) and presence of DPN, and AAR was positively associated with VPT and presence of DPN independent of sex, age, body mass index, and diabetic duration (P<0.01 or P<0.05). Moreover, AAR remained significantly associated with a higher odds ratio (OR) of DPN (OR 2.413, 95% confidence interval [CI] 1.081-5.386, P<0.05) after multivariate adjustment. Additionally, the risk of presence of DPN increased progressively as AAR quartiles increased (all P for trend <0.01) in both male and female subjects, and the highest quartile of AAR of male and female subjects was respectively associated with 107.3% (95% CI: 1.386-3.101; P<0.01) and 136.8% (95% CI: 1.550-3.618; P<0.01) increased odds of DPN compared with the lower quartiles. Last, the analysis of receiver operating characteristic curves revealed that the best cutoff values for AAR to predict the presence of DPN were 0.906 (sensitivity: 70.3%; specificity: 49.2%; and area under the curve [AUC]: 0.618) and 1.402 (sensitivity: 38%; specificity: 81.9%; and AUC: 0.600) in male and female subjects, respectively.

**Conclusions:**

These findings suggest that the high AAR may be associated with the presence of DPN in Chinese patients with T2DM, and may be used as an additional indicator of risk of DPN.

## Introduction

Diabetic peripheral neuropathy (DPN) is the most common but usually underestimated chronic microvascular complication that first present in the distal extremities and can result in either numbness or chronic pain; and is a major risk factor for Charcot joints, diabetic foot ulcers (DFU), and limb amputation in diabetic patients (1, 2). DPN has now been considered an increasing public health problem, owing to its close association with considerable morbidity and mortality, heavy economic burden, and compromised quality of life (1, 3). However, the current treatment for DPN involves only symptomatic relief, and often the results are disappointing. Therefore, it is urgent to find an indicator for screening the high-risk population of DPN, resulting in early identification and, consequently, early intervention.

A number of studies have shown that type 2 diabetes mellitus (T2DM) is an independent risk factor for the development of nonalcoholic fatty liver disease (NAFLD) and progression to liver fibrosis and cirrhosis (4, 5). Also, NAFLD and liver fibrosis have been reported to play an important role in the presence and progression of DPN (6–8). Alanine aminotransferase (ALT) and aspartate aminotransferase (AST) were the two most common liver enzymes that reflect hepatocellular injury and death, and liver function. The concept of AST/ALT ratio (AAR) that represents the simultaneous alteration of AST and ALT levels was first put forwarded by De Ritis in 1957 (9). Since then, AAR has been reported to be a widely used liver fibrosis marker, and in addition, an established predictive marker of liver fibrosis severity in patients with liver disease and other non-hepatic diseases (10, 11). Besides, AAR was correlated with oxidative stress, systemic inflammation, and insulin resistance (IR) (12, 13), and implicated in the incidence and development of a wider range of cardiometabolic diseases, including metabolic syndrome (MetS) and its components including obesity, hyperglycemia or T2DM, hypertension, and hyperlipidemia, NAFLD as a hepatic manifestation of MetS, peripheral artery disease (PAD), arteriosclerosis, arterial stiffness, stroke, and cardiovascular diseases (CVD) (14–19), all of which have been proved to be closely associated with diabetic microvascular complications (20–22). Considering the strong interrelationship between diabetic microvascular complications and above-mentioned cardiometabolic diseases and the important role of liver fibrosis in diabetic microvascular complications (7, 23, 24), it is reasonable to hypothesize that T2DM individuals with high AAR would have a high risk for diabetic microvascular complications. Indeed, two clinical studies suggested that high AAR was an independent risk factor for diabetic nephropathy (DN), and was associated with more severe renal pathologic lesions and worse renal function (12, 25). As far as we are aware, the relationship of the AAR with DPN, however, has never been determined, and the underpinning mechanisms are less well understood.

Therefore, this cross-sectional study was conducted to investigate the relationship between AAR and risk of presence of DPN in Chinese adults with T2DM. Moreover, the possible mechanisms were explored by analyzing the potential relationships among AAR and metabolic and vascular parameters, and inflammation and oxidative stress markers.

## Methods

### Study population

A total of 3514 confirmed or newly diagnosed T2DM inpatients aged 18–89 years between August 2012 and September 2015, who were admitted to the Department of Endocrinology at the Affiliated Hospital of Southwest Medical University for screening of diabetic chronic complications and to optimize their anti-diabetic regimen, were initially recruited. T2DM was diagnosed based on the 1999 World Health Organization criteria (26). Subjects were excluded if they had any of the following criteria: 1) other types of diabetes other than T2DM, severe DFU (grades III-V according to the Wagner classification) or previous amputation, recent acute complications of diabetes, including diabetic ketoacidosis, hyperglycemic hyperosmolar state, hyperosmolar coma and hypoglycemia; 2) endocrine diseases other than T2DM, such as thyroid disease, parathyroid disease, adrenal diseases, pituitary diseases; 3) presence of non-diabetes-related neuropathy such as chronic inflammatory demyelinating polyneuropathy, cervical and lumbar diseases, and severe cerebrovascular disease (ischemic and haemorrhagic stroke); 4) severe respiratory disease, congestive heart failure (New York Heart Association functional class IV), severe renal failure (estimated glomerular filtration rate (eGFR) <30 mL/min/1.73 m^2^), hematological diseases, thromboembolic disease; 5) autoimmune or viral hepatitis, alcohol-induced or drug-induced liver disease, cholestatic or metabolic/genetic liver disease, liver cirrhosis, and other chronic liver disease, gall bladder and biliary tract diseases; 6) connective tissue, inflammatory and recent active infectious disease, and stress conditions, autoimmune diseases; 7) history of malignancies and mental illness; 8) alcoholism; 9) pregnancy and lactation; 10) use of immunosuppressive agents, antioxidants, anti-inflammatory, antibiotics, analgesics, systemic corticosteroids, multivitamins or vitamin B12 supplements; 11) use of possible or known drugs affecting peripheral nerve function and sympathetic system; 12) missing or incomplete demographic or clinical characteristic data. After applying the exclusion criteria, 1562 participants aged 18-89 years were eligible and finally enrolled in the cross-sectional study.

The study was reviewed and approved by the human research ethics committee of the Affiliated Hospital of Southwest Medical University, and was performed in accordance with the Helsinki Declaration. All patients gave informed consent before participating in this study.

### Data collection and measurements

During face-to-face interviews, trained interviewers administered a detailed standardized questionnaire, which consisted of information on their demographic characteristics (sex, age), lifestyle characteristics (physical activity, smoking and drinking status, etc.), personal medical history (hypertension, coronary heart disease (CHD), DFU, PAD, diabetic retinopathy (DR), DN, NAFLD, and other diseases), disease duration, family history, as detailed elsewhere. Then, all patients with T2DM received anthropometric examination, physical examination, laboratory tests, and evaluation of diabetes-related complications.

Body weight and height were measured by trained interviewers under standardized conditions following a standardized protocol, and body mass index (BMI) was calculated as body weight (kg) divided by the square of the height (m). Systolic blood pressures (SBP) and diastolic blood pressures (DBP) were measured in all subjects on the right arm using a standard mercury sphygmomanometer (27).

Venous blood samples were gathered from each participant in the morning after an overnight fast (at least 8 h) for measurement of fasting blood glucose (FBG), glycated hemoglobin A1C (HbA1c), total cholesterol (TC), triglyceride (TG), high density lipoprotein cholesterol (HDL-C), low-density lipoprotein cholesterol (LDL-C), apolipoprotein A (apoA), apolipoprotein B (apoB), AST, ALT, total bilirubin (TBIL), glutamyl transpeptidase (GGT), serum albumin (ALB), creatinine (Cr), uric acid (UA), white blood cell (WBC), neutrophil, and lymphocyte counts, red blood cell distribution width (RDW), and fibrinogen according to relevant protocols and guidelines at the registered central laboratory located at the Affiliated Hospital of Southwestern Medical University, which is accredited in line with the international organization for standardization (ISO) 15189 standard for quality management specific to medical laboratories.

Triglyceride-glucose (TyG) index was calculated using the following equation: ln (fasting TG [mg/dL] × FBG [mg/dL]/2) (28). The atherogenic index of plasma (AIP) was calculated as ln (TG/HDL-C) and the atherogenic coefficient (AC) was calculated as (TC-HDL-C/HDL-C) (29). Hepatic steatosis index (HSI) was defined as follows: HSI = 8 × ALT/AST ratio + BMI (+2, if diabetes; +2, if female) (30). The AAR was calculated as AST/ALT ratio. Neutrophil to lymphocyte ratio (NLR) was calculated by dividing the neutrophil count by lymphocyte count. The eGFR was evaluated with the Chronic Kidney Disease Epidemiology Collaboration (CKD-EPI) equations modified by a Japanese coefficient (31, 32). Urinary albumin and Cr were measured from three fresh morning spot urine sample on three separate occasions within 6 months. Urinary albumin was measured with immunoturbidimetric tests. Urinary Cr was measured enzymatically. The urinary albumin-to- Cr ratio (ACR; mg/g creatinine) was calculated by dividing urinary albumin by urinary Cr (33, 34). Patients were then classified as having DN if they had an eGFR < 60 mL/min/1.73m^2^ and/or an ACR > 30 mg/g in two out of three random voided urine samples (32–34).

### Foot examination and definition of DPN, PAD, and DFU

All patients with T2DM were asked whether they had numbness, pain (prickling or stabbing, shooting, burning or aching pain), and paresthesia (abnormal cold or heat sensation, allodynia and hyperalgesia) in the toes, feet, legs or upper-limb. Then, an experienced physician performed the neurologic examination which included vibration, light touch, and achilles tendon reflexes on both sides in the knee standing position (as being either presence or weakening or loss). Vibration perception threshold (VPT) was assessed at the metatarsophalangeal joint dig I using a neurothesiometer (Bio- Thesiometer; Bio-Medical Instrument Co., Newbury, OH, USA). First, the patients were informed how to know the vibration sensation is felt by gradually turning the amplitude from zero to maximum, then the test began again from zero and they were asked to say the moment that they first felt it. Measurements were made on the planter aspect of the big toe bilaterally, three times consecutively for each big toe. The median of three readings is accepted as the VPT value of that measurement (35). Sensitivity to touch was also tested using a 5.07/10-g Semmes-Weinstein monofilament (SWM) at four points on each foot: three on the plantar and one on the dorsal side. The 10-g SWM was placed perpendicular to the skin and pressure was applied until the filament just buckled with a contact time of 2 s. Inability to perceive the sensation at any one site was considered abnormal (36, 37). DPN was defined as VPT ≥25 V and/or inability to feel the monofilament (35), and then participants were divided into DPN group and no DPN group.

Ankle brachial index (ABI) was measured noninvasively by a continuous-wave Doppler ultrasound probe (Vista AVS, Summit Co., USA) with participants in the supine position after at least 5 min of rest. Leg-specific ABI was calculated by dividing the higher SBP in the posterior tibial or dorsalis pedis by the higher of the right or left brachial SBP (33, 38). Patients were diagnosed as having PAD if an ABI value <0.9 on either limb (33, 38).

DFU was defined as ulceration of the foot (distally from the ankle and including the ankle) associated with neuropathy and different grades of ischemia and infection (39).

### Other classifications and definitions

A Canon CR-2 Digital Retinal Camera was performed to obtain two-field fundus photography of patient’s eyes (Canon Inc., Kanagawa, Japan). The presence of DR was assessed by high-quality fundus photographs and an ophthalmologist. NAFLD diagnosis was based on the detection of hepatic steatosis by abdominal ultrasound while excluding drugs, viruses, or alcohol as the cause (40)**. **MetS was defined according to Chinese Diabetes Society (CDS) criteria (41) if they have three or more of the following risk factors (1): overweight or obese (BMI ≥ 25.0 kg/m^2^) (2); hyperglycemia (FBG ≥ 6.1 mmol/l and/or 2-hour postprandial plasma glucose ≥ 7.8 mmol/l, or under treatment for diabetes) (3); hypertension (SBP ≥ 140 mmHg, DBP ≥ 90 mmHg, or on antihypertensive medication); and (4) dyslipidemia, defined as TG ≥ 1.7 mmol/l and/or HDL-C < 0.9 mmol/l (men) or <1.0 mmol/l (women). CHD was defined as a positive history of myocardial infarction, bypass operation, a diagnostic finding in angiography or positive exercise test (42).

### Statistical analysis

Statistical analyses were conducted using the Statistical Package for Social Sciences (SPSS) (version 20.0; IBM, Chicago, IL). All data were first analyzed for normality of distribution using the Kolmogorov–Smirnov test of normality, and homogeneity of variance using the Levene homogeneity of variance test. Continuous data are presented as mean ± standard deviation (SD), and categorical data are presented as absolute and relative frequencies (n, %).

All patients with T2DM were placed into four groups according to AAR quartiles: quartile (Q) 1 group, 0.32–0.80; Q2 group, 0.81–1.00; Q3 group, 1.01–1.27; and Q4 group, 1.28-5.26. Meanwhile, male and female patients were divided into four quartile groups by AAR level, respectively: Q1 group (male: 0.32-0.75; female: 0.36–0.88), Q2 group (male: 0.76–0.94; female: 0.89–1.08), Q3 group (male: 0.95–1.19; female: 1.09–1.34), and Q4 group (male: 1.20– 3.98; female: 1.35– 5.26). Continuous variables were compared by Student’s t test and one-way analysis of variance (ANOVA), whereas skewed distribution variables were compared by Mann-Whitney U and Kruskal-Wallis tests. Categorical variables were compared across groups using χ^2^ tests. As AAR was non-normally distributed, Spearman correlation coefficients were performed to assess whether there was an association between AAR and other variables, and the partial correlation coefficient was also used to control for the effects of age, sex, BMI, and diabetic duration. The collinearity diagnostics analysis in linear regression models was also performed to assess whether multiple collinearity exists in these independent variables. The associations of AAR and other variables with the risk of presence of DPN in all T2DM patients were explored by the univariable logistic regression analysis, and then determined using a multivariable logistic regression analysis with those variables achieving *P* ≤ 0.20 in our univariable analysis entered into this model. Further, binary logistic regression analyses were conducted to investigate the association of AAR quartiles with the risk of presence of DPN in all subjects, male subjects, and female subjects, and odd ratio (OR) and 95% confidence interval (CI) were estimated. Possible dose-response relationships between AAR and DPN were examined by the trend test. Last, the predictive validity of AAR for the presence of DPN was determined using receiver operating characteristic (ROC) curves and area under the curve (AUC) in all subjects, male subjects, and female subjects.

Results were considered to be statistically significant at a P value <0.05.

## Results

### Clinical and laboratory characteristics of study participants

The clinical and laboratory characteristics of 1562 patients with T2DM (774 male, 49.55%, and 788 female, 50.45%) according to AAR quartiles were summarized in **Table 1**. Overall, mean age was 59.74 years, BMI was 24.19 kg/m^2^, diabetic duration was 7.55 years, and AAR was 1.10. Patients with higher AAR quartiles tended to be female and relatively older, less user of smoking, and have longer diabetic duration, higher levels of SBP, HDL-C, apoA, AST, AAR, RDW, serum Cr, urinary ACR, VPT, presence of DPN, DN, hypertension, DFU, PAD, and lower BMI, DBP, TG, LDL-C, apoB, TyG, FBG, HbA1c, ALT, TBIL, GGT, serum ALB, lymphocyte counts, eGFR, ABI, HSL, prevalence of dyslipidemia, NAFLD, and MetS compared to those with lower quartiles (P<0.01 or P<0.05). **Supplementary Table 1** reported characteristics of all T2DM patients by DPN. Patients with DPN had significantly older age, longer diabetic duration, higher SBP, FBG, HbA1c, AAR, WBC, neutrophil counts, NLR, fibrinogen, serum Cr, urinary ACR, VPT, prevalence of DN, DR, hypertension, CHD, DFU, PAD, and lower BMI, DBP, TC, TG, apoA, ALT, AST, TBIL, GGT, serum ALB, lymphocyte counts, eGFR, ABI, HSL, and prevalence of NAFLD than those without DPN (P<0.01 or P<0.05).

**Table 1:** Characteristics of study participants according to AAR quartiles.

Variable	Total	Q1	Q2	Q3	Q4	*P*
	(n=1562)	(n=394)	(n=387)	(n=386)	(n=395)	
		0.32–0.80	0.81–1.00	1.01–1.27	1.28– 5.26	value
Male (n, %)	774 (49.55%)	252 (63.96%)	195 (50.39%)	182 (47.15%)	145 (36.71%)	0.000
Age (years)	59.74 ± 11.32	54.79 ± 11.32	59.33 ± 10.46	60.89 ± 10.72	63.94 ± 10.80	0.000
BMI (kg/m^2^)	24.19 ± 3.66	24.78 ± 3.66	24.69 ± 3.49	24.21 ± 3.60	23.08 ± 3.64	0.000
Diabetic duration (years)	7.55 ± 6.45	5.10 ± 5.09	7.76 ± 6.18	8.09 ± 6.42	9.27 ± 7.22	0.000
Smoking (n, %)	333 (21.32%)	120 (30.46%)	72 (18.60%)	73 (18.91%)	68 (17.22%)	0.000
SBP (mmHg)	132.48 ± 20.82	129.16 ± 18.94	132.97 ± 20.94	133.19 ± 22.03	134.60 ± 20.98	0.001
DBP (mmHg)	72.06 ± 12.17	74.50 ± 12.31	72.81 ± 11.05	71.34 ± 12.72	69.61 ± 12.05	0.000
TC(mmol/L)	4.86 ± 1.35	4.88 ± 1.28	4.83 ± 1.27	5.00 ± 1.46	4.75 ± 1.37	0.086
TG (mmol/L)	2.36 ± 2.60	2.67 ± 2.57	2.42 ± 2.00	2.42 ± 3.59	1.92 ± 1.82	0.000
HDL-C (mmol/L)	1.18 ± 0.37	1.08 ± 0.30	1.14 ± 0.33	1.20 ± 0.40	1.29 ± 0.40	0.000
LDL-C (mmol/L)	2.78 ± 1.00	2.78 ± 0.94	2.75 ± 0.96	2.91 ± 1.09	2.69 ± 1.02	0.025
ApoA (g/L)	1.33 ± 0.30	1.28 ± 0.26	1.33 ± 0.28	1.35 ± 0.32	1.36 ± 0.35	0.001
ApoB (g/L)	0.91 ± 0.29	0.94 ± 0.26	0.90 ± 0.26	0.93 ± 0.30	0.87 ± 0.33	0.000
TyG	9.48 ± 1.08	9.67 ± 1.11	9.61 ± 0.92	9.46 ± 1.04	9.17 ± 1.15	0.000
AIP	0.48 ± 0.02	0.67 ± 0.04	0.57 ± 0.04	0.46 ± 0.05	0.20 ± 0.04	0.000
AC	3.52 ± 2.42	3.88 ± 2.46	3.61 ± 2.26	3.66 ± 3.09	2.95 ± 1.53	0.000
FBG (mmol/L)	10.87 ± 5.20	11.68 ± 5.20	11.00 ± 4.88	10.52 ± 5.08	10.28 ± 5.50	0.000
HbA1c (%)	9.52 ± 2.50	9.96 ± 2.47	9.55 ± 2.30	9.38 ± 2.50	9.20 ± 2.68	0.000
ALT (U/L)	23.15 ± 17.53	38.09 ± 22.46	23.11 ± 10.58	17.92 ± 12.20	13.40 ± 10.66	0.000
AST (U/L)	21.86 ± 15.81	24.20 ± 12.88	20.98 ± 9.63	20.15 ± 13.48	22.08 ± 23.39	0.000
AAR	1.10 ± 0.46	0.66 ± 0.10	0.91 ± 0.06	1.13 ± 0.08	1.68 ± 0.50	0.000
TBIL (μmol/L)	12.27 ± 5.62	13.11 ± 6.52	12.61 ± 5.37	12.10 ± 5.39	11.24 ± 4.94	0.000
GGT (U/L)	43.74 ± 2.53	55.57 ± 7.57	41.92 ± 2.66	40.50 ± 4.84	36.86 ± 3.70	0.048
Serum ALB (g/L)	40.97 ± 4.91	41.79 ± 4.48	41.76 ± 4.42	41.08 ± 4.77	39.32 ± 5.48	0.000
WBC (*10^9^/L)	6.81 ± 2.41	7.01 ± 2.40	6.64 ± 1.85	6.64 ± 2.12	6.94 ± 3.07	0.119
Neutrophil (*10^9^/L)	4.60 ± 2.27	4.73 ± 2.22	4.40 ± 1.66	4.43 ± 1.92	4.82 ± 3.02	0.264
Lymphocyte (*10^9^/L)	1.65 ± 0.62	1.73 ± 0.64	1.66 ± 0.61	1.63 ± 0.59	1.57 ± 0.64	0.006
NLR	3.36 ± 0.08	3.22 ± 0.14	3.11 ± 0.11	3.16 ± 0.12	3.94 ± 0.22	0.192
RDW (%)	13.17 ± 1.28	13.17 ± 1.37	13.13 ± 1.21	13.01 ± 1.19	13.35 ± 1.32	0.013
Fibrinogen(g/L)	3.68 ± 1.35	3.49 ± 1.25	3.62 ± 1.34	3.71 ± 1.29	3.86 ± 1.47	0.060
Serum UA (μmol/L)	316.52 ± 108.40	321.33 ± 113.45	313.86 ± 95.81	316.39 ± 108.38	314.45 ± 115.00	0.908
Serum Cr (μmol/L)	73.74 ± 47.41	69.31 ± 40.69	71.05 ± 39.74	74.87 ± 49.83	79.69 ± 56.72	0.008
eGFR (mL/min/1.73 m^2^)	91.91 ± 26.12	100.40 ± 24.34	93.29 ± 25.12	89.90 ± 25.32	84.07 ± 27.00	0.000
Urinary ACR (mg/g)	238.78 ± 22.07	140.82 ± 35.57	177.12 ± 33.57	209.69 ± 32.92	427.31 ± 64.97	0.000
ABI	1.02 ± 0.15	1.04 ± 0.12	1.03 ± 0.15	1.01 ± 0.16	1.00 ± 0.18	0.003
VPT (V)	16.40 ± 9.90	14.65 ± 9.47	15.42 ± 8.96	16.49 ± 9.17	19.03 ± 11.28	0.000
HSL	33.46 ± 5.45	37.92 ± 4.65	34.45 ± 3.82	32.23 ± 4.25	29.14 ± 4.82	0.000
Dyslipidemia (n, %)	844 (54.03%)	245 (62.18%)	219 (56.59%)	211 (54.66%)	169 (42.78%)	0.000
NAFLD (n, %)	713 (45.65%)	229 (58.12%)	204 (52.71%)	159 (41.19%)	121 (30.63%)	0.000
MetS (n, %)	740 (47.38%)	191 (48.48%)	193 (49.87%)	196 (50.78%)	160 (40.51%)	0.015
Microvascular complications						
DN (n, %)	657 (42.06%)	121 (30.71%)	150 (38.76%)	169 (43.78%)	217 (54.94%)	0.000
DR (n, %)	201 (12.87%)	46 (11.68%)	43 (11.11%)	58 (15.03%)	54 (13.67%)	0.335
DPN (n, %)	236 (15.11%)	43 (10.91%)	51 (13.18%)	55 (14.25%)	87 (22.03%)	0.000
Macrovascular complications						
Hypertension (n, %)	828 (53.01%)	180 (45.69%)	206 (53.23%)	213 (55.18%)	229 (57.97%)	0.004
CHD (n, %)	140 (8.96%)	24 (6.09%)	39 (10.08%)	40 (10.36%)	37 (9.37%)	0.134
DFU (n, %)	115 (7.36%)	19 (4.82%)	24 (6.20%)	31 (8.03%)	41 (10.38%)	0.018
PAD (n, %)	154 (9.86%)	26 (6.60%)	28 (7.24%)	40 (10.36%)	60 (15.19%)	0.000

Data are mean ± SD. SD, standard deviation; Q, quartile; BMI, body mass index; SBP, systolic blood pressure; DBP, diastolic blood pressure; TC, total cholesterol; TG, triglyceride; HDL-C, high-density lipoprotein cholesterol; LDL-C, low-density lipoprotein cholesterol; apoA, apolipoprotein A; apoB, apolipoprotein B; TyG, triglyceride-glucose; AIP, atherogenic index of plasma; AC, atherogenic coefficient; FBG, fasting blood glucose; HbA1c, glycated hemoglobin A1c; ALT, alanine aminotransferase; AST, aspartate aminotransferase; AAR, aminotransferase to alanine aminotransferase ratio; TBIL, total bilirubin; GGT, gamma-glutamyl transferase; ALB, albumin, WBC, white blood cell; NLR, neutrophil to lymphocyte ratio; RDW, red blood cell distribution width; UA, uric acid; Cr, creatinine; eGFR, estimated glomerular filtration rate; ACR, albumin- to-creatinine ratio; ABI, ankle-brachial index; VPT, vibration perception threshold; DPN, diabetic peripheral neuropathy; HSL, hepatic steatosis index; NAFLD, nonalcoholic fatty liver disease; MetS, metabolic syndrome; DN, diabetic nephropathy; DR, diabetic retinopathy; DPN, diabetic peripheral neuropathy; CHD, coronary heart disease; DFU, diabetic foot ulceration; PAD, peripheral arterial disease.

### Association of AAR with clinical and laboratory characteristics in study subjects

**Table 2** showed the association of AAR with clinical and laboratory characteristics in all patients with T2DM performed by Spearman and partial correlation coefficient. The results revealed that AAR was positively associated with age, sex distribution, diabetic duration, SBP, HDL-C, apoA, fibrinogen, serum Cr, urinary ACR, VPT and prevalence of DPN, DN, hypertension, DFU, PAD, and negatively with BMI, smoking, DBP, TG, apoB, TyG, FBG, HbA1c, ALT, AST, TBIL, GGT, serum ALB, WBC, lymphocyte counts, eGFR, ABI, HSL, and prevalence of dyslipidemia, NAFLD and MetS (P<0.01 or P<0.05). After adjustments for sex, age, BMI, and diabetic duration, the associations among HbA1c, ALT, serum ALB, eGFR, urinary ACR, VPT, HSL, presence of dyslipidemia, NAFLD, DPN, DN, PAD and AAR were attenuated but remained statistically significant (P<0.01 or P<0.05).

**Table 2:** Association between AAR and clinical and laboratory characteristics in study subjects.

	*r*	*P*-value	Adjusted *r*	Adjusted *P*-value
Age	0.305	0.010	–	–
Sex (female vs male)	0.197	0.000	–	–
BMI	-0.185	0.000	–	–
Diabetic duration	0.226	0.000	–	–
Smoking	-0.105	0.000	0.027	0.295
SBP	0.099	0.000	0.029	0.496
DBP	-0.166	0.000	-0.054	0.214
TC	-0.034	0.177	-0.017	0.690
TG	-0.170	0.000	-0.034	0.425
HDL-C	0.207	0.000	0.060	0.162
LDL-C	-0.029	0.249	-0.004	0.929
ApoA	0.091	0.000	-0.008	0.846
ApoB	-0.100	0.000	0.041	0.344
TyG	-0.201	0.000	-0.084	0.053
AIP	-0.208	0.000	-0.051	0.235
AC	-0.199	0.000	-0.032	0.461
FBG	-0.154	0.000	-0.060	0.161
HbA1c	-0.145	0.000	-0.086	0.047
ALT	-0.703	0.000	-0.380	0.000
AST	-0.138	0.000	0.038	0.384
TBIL	-0.131	0.000	-0.060	0.161
GGT	-0.324	0.000	0.053	0.220
Serum ALB	-0.181	0.000	-0.130	0.003
WBC	-0.052	0.039	0.035	0.423
Neutrophil	-0.037	0.148	0.044	0.303
Lymphocyte	-0.085	0.001	-0.066	0.128
NLR	0.044	0.086	0.082	0.057
RDW	0.047	0.064	0.081	0.060
Fibrinogen	0.086	0.017	0.055	0.205
Serum UA	-0.020	0.440	0.039	0.371
Serum Cr	0.087	0.001	0.068	0.114
eGFR	-0.258	0.000	-0.112	0.010
Urinary ACR	0.192	0.000	0.105	0.014
ABI	-0.098	0.000	-0.050	0.251
VPT	0.209	0.000	0.110	0.011
HSL	-0.665	0.000	-0.807	0.000
Dyslipidemia	-0.141	0.000	-0.068	0.009
NAFLD	-0.215	0.000	-0.131	0.000
MetS	-0.060	0.017	0.016	0.530
DPN	0.124	0.000	0.068	0.008
DN	0.186	0.000	0.118	0.000
DR	0.030	0.238	0.006	0.817
Hypertension	0.087	0.001	0.007	0.785
CHD	0.046	0.072	-0.002	0.926
DFU	0.084	0.001	0.043	0.104
PAD	0.129	0.000	0.052	0.048

### Univariate and multivariate analysis of determinants of DPN in study subjects

**Table 3** displayed the associations of AAR and other variables with the risk of presence of DPN. The univariate logistic regression analysis revealed that age, BMI, diabetic duration, SBP, DBP, TC, TG, apoA, FBG, HbA1c, ALT, AST, AAR, TBIL, serum ALB, WBC, neutrophil and lymphocyte counts, NLR, fibrinogen, serum Cr, eGFR, urinary ACR, ABI, HSL, and prevalence of NAFLD, DN, DR, hypertension, CHD, DFU, PAD were significantly associated with the presence of DPN (P<0.01 or P<0.05).Multivariable logistic regression analysis showed that age, TyG, AAR, serum ALB, and DFU were significantly and independently associated with the presence of DPN (P<0.01 or P<0.05). Notably, each SD increase in AAR was associated with a significant 2.413-fold increased odds of DPN (95% CI, 1.081-5.386, P<0.05).

**Table 3:** Binary logistic regression analyses of variables contributing to DPN in patients with T2DM.

Variables	Univariate analysis			Multivariate analysis		
	B	OR (95%CI)	*P*-value	B	OR (95%CI)	*P*-value
Sex (female vs male)	-0.221	0.802 (0.607-1.058)	0.119			
Age	0.072	1.075 (1.059-1.090)	0.000	0.040	1.041 (1.004-1.080)	0.029
BMI	-0.059	0.943 (0.905-0.982)	0.005			
Diabetic duration	0.068	1.071 (1.050-1.092)	0.000			
Smoking	-0.101	0.904 (0.640-1.277)	0.568			
SBP	0.007	1.007 (1.000-1.014)	0.038			
DBP	-0.017	0.983 (0.971-0.994)	0.004			
TC	-0.149	0.862 (0.769-0.966)	0.011			
TG	-0.175	0.839 (0.758-0.930)	0.001			
HDL-C	0.040	1.041 (0.710-1.525)	0.838			
LDL-C	-0.040	0.961 (0.834-1.107)	0.579			
ApoA	-0.822	0.440 (0.269-0.718)	0.001			
ApoB	-0.030	0.970 (0.596-1.579)	0.903			
TyG	-0.119	0.888 (0.786-1.002)	0.054	2.156	8.639 (1.036-72.058)	0.046
AC	-0.129	0.879 (0.803-0.962)	0.005			
FBG	0.027	1.028 (1.002-1.053)	0.032			
HbA1c	0.103	1.109 (1.051-1.169)	0.000			
ALT	-0.031	0.970 (0.957-0.982)	0.000			
AST	-0.030	0.971 (0.954-0.987)	0.001			
AAR	0.667	1.949 (1.497-2.537)	0.000	0.881	2.413 (1.081-5.386)	0.032
TBIL	-0.058	0.944 (0.916-0.973)	0.000			
GGT	-0.001	0.999 (0.997-1.001)	0.381			
Serum ALB	-0.151	0.860 (0.835-0.886)	0.000	-0.102	0.903 (0.843-0.967)	0.004
WBC	0.101	1.106 (1.052-1.162)	0.000			
Lymphocyte	-0.767	0.464 (0.355-0.608)	0.000			
NLR	0.111	1.117 (1.074-1.163)	0.000			
RDW	0.060	1.062 (0.958-1.177)	0.256			
Fibrinogen	0.409	1.505 (1.328-1.704)	0.000			
Serum UA	0.001	1.001 (1.000-1.002)	0.127			
Serum Cr	0.006	1.006 (1.004-1.008)	0.000			
eGFR	-0.023	0.978 (0.973-0.983)	0.000			
Urinary ACR	0.678	1.969 (1.613-2.404)	0.000			
ABI	-3.087	0.046 (0.021-0.098)	0.000			
HSL	-0.060	0.941 (0.917-0.966)	0.000			
Dyslipidemia	-0.191	0.826 (0.626-1.090)	0.178			
NAFLD	-0.426	0.653 (0.491-0.869)	0.003			
MetS	0.144	1.155 (0.875-1.523)	0.309			
DN	1.035	2.814 (2.111-3.751)	0.000			
DR	0.738	2.092 (1.466-2.984)	0.000			
Hypertension	0.490	1.632 (1.227-2.171)	0.001			
CHD	0.958	2.606 (1.757-3.865)	0.000			
DFU	1.817	6.153 (4.133-9.159)	0.000	1.337	3.807 (1.753-8.267)	0.001
PAD	1.632	5.117 (3.575-7.324)	0.000			

Beta is the standardized coefficient and measures the influence of each variables on DPN; OR is the odds ratio and refers to the risk of DPN.

### Association of AAR quartiles with the risk of presence of DPN in study subjects

As shown in **Table 4**, the risk of presence of DPN also increased progressively as AAR quartiles increased in all subjects, male subjects, and female subjects, respectively (all P for trend <0.01). When compared to the lower quartiles (Q1, Q2, and Q3), the highest quartile of AAR (Q4) of all subjects, male subjects, and female subjects were significantly associated with increased odds for DPN (OR = 1.930, 2.073, and 2.368, respectively). Even per SD increase in AAR of all subjects, male subjects, and female subjects were respectively associated with were more likely to have DPN (OR = 1.358, 1.416, and 1.348, respectively).

**Table 4:** Association between quartiles of AAR and risk of presence of DPN in study participants.

AAR	DPN	
	Odds ratio (95% CI)	*P*
All subjects		
Per SD increase	1.358 (1.204-1.533)	0.000
Quartiles of AAR		
Q1 (0.32–0.80)	1 (reference)	
Q2 (0.81–1.00)	1.239 (0.804-1.909)	0.331
Q3 (1.01–1.27)	1.356 (0.886-2.077)	0.161
Q4 (1.28– 5.26)	2.306 (1.552-3.426)	0.000
*P* for trend	0.000	
Q4 versus. Q1, Q2, Q3	1.930 (1.439-2.588)	0.000
	Male subjects	
Per SD increase	1.416 (1.198-1.674)	0.000
Quartiles of AAR		
Q1 (0.32–0.75)	1 (reference)	
Q2 (0.76–0.94)	1.450 (0.774-2.717)	0.246
Q3 (0.95–1.19)	2.115 (1.163-3.849)	0.014
Q4 (1.20– 3.98)	3.101 (1.746-5.510)	0.000
*P* for trend	0.000	
Q4 versus. Q1, Q2, Q3	2.073 (1.386-3.101)	0.000
	Female subjects	
Per SD increase	1.348 (1.133-1.603)	0.001
Quartiles of AAR		
Q1 (0.36–0.88)	1 (reference)	
Q2 (0.89–1.08)	1.071 (0.572-2.004)	0.831
Q3 (1.09–1.34)	0.945 (0.498-1.793)	0.864
Q4 (1.35– 5.26)	2.379 (1.366-4.145)	0.002
*P* for trend	0.002	
Q4 versus. Q1, Q2, Q3	2.368 (1.550-3.618)	0.000

Data are expressed as OR (95% CI) +P value, unless stated otherwise. OR, odds ratio; CI, confidence interval.

### Predictive value of AAR in screening for the presence of DPN in T2DM patients

**Figure 1:**
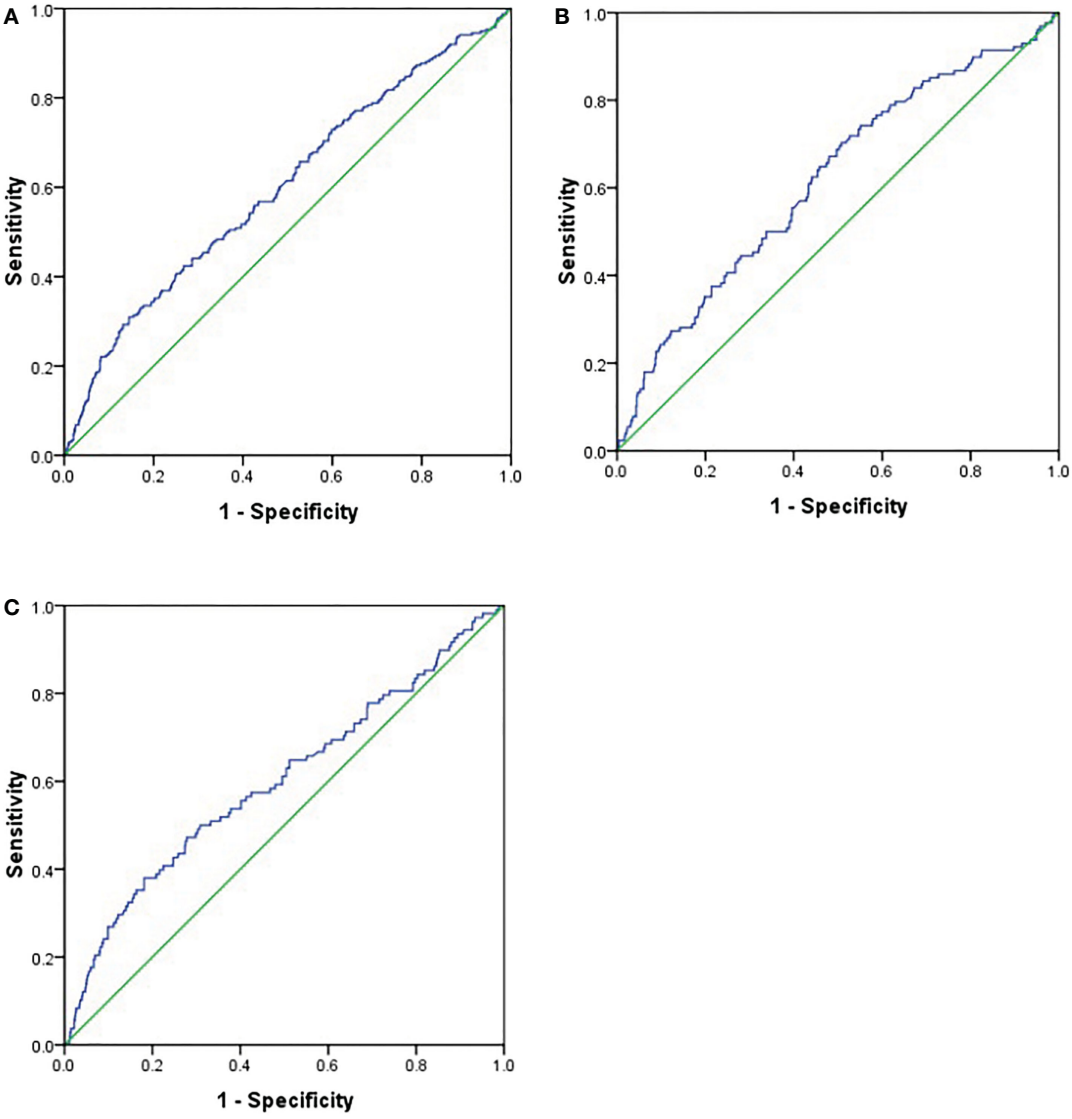
Receiver operating characteristics (ROC) curve analysis of aminotransferase to alanine aminotransferase ratio (AAR) to inidicate DPN. **(A)**. all subjects; **(B)**. male subjects; **(C)**. female subjects.

To explore the predictive value of AAR for DPN, we analyzed the ROC curves of AAR. The results revealed that the best cutoff value for AAR to predict the presence of DPN was 1.40 (sensitivity: 30.90%; specificity: 85.50%; and AUC: 0.600; **Figure 1A**) in all subjects, and the best cutoff values for AAR to predict the presence of DPN were 0.906 (sensitivity: 70.3%; specificity: 49.2%; and AUC: 0.618; **Figure 1B**) and 1.402 (sensitivity: 38%; specificity: 81.9%; and AUC: 0.600; **Figure 1C**) in male and female subjects, respectively.

## Discussion

To our knowledge, this was the first study to investigate the relationship between AAR and risk of presence of DPN. We found that patients with higher AAR quartiles had higher presence of DPN, and AAR was an independent determinant of presence of DPN after multivariate adjustment. Additionally, the risk of presence of DPN increased progressively as AAR quartiles increased in both sexes. Last, the analysis of ROC curves revealed that AAR could predict the presence of DPN in both sexes. These findings suggest that high AAR may be associated with the presence of DPN in hospitalized Chinese T2DM patients, and may be used as an additional indicator of risk of DPN.

As mentioned earlier, AAR, an emerging indicator of liver function, has been reported to effectively predict the severity of liver fibrosis in patients with various liver disease including NAFLD (10, 11). There is now growing evidence that NAFLD is more common and often advanced in patients with T2DM, easily progressing to nonalcoholic steatohepatitis and advanced liver fibrosis, than in the general population (6, 43–46). Considering a certain intrinsic correlation among AAR, NAFLD and liver fibrosis, and diabetic vascular complication (6, 10, 11, 15–19, 43–46), it is plausible that AAR may be associated with the presence of DPN, and high AAR may be an early signal for being at risk for DPN. In the present study, we found that patients with higher AAR quartiles tended to have higher VPT, a widely recommended indicator of the presence and severity of confirmed clinical neuropathy (47), and similarly, patients with DPN had significantly higher AAR than those without. Moreover, AAR was positively associated with VPT and presence of DPN. Altogether, these data preliminarily argue that there was a potential relationship between AAR and the presence and severity of DPN. Besides, AAR was significantly and independently associated with the presence of DPN after multivariate adjustment. Additionally, the risk of presence of DPN increased progressively as AAR quartiles increased in both sexes. More importantly, AAR could predict the presence of DPN in both sexes. These data were broadly similar to the findings of previous studies showing that noninvasive biomarkers of liver fibrosis, such as NAFLD fibrosis score and fibrosis-4 score were independently associated with DPN (6, 8, 48, 49), further suggesting that higher AAR, another novel liver fibrosis marker, could be linked to an increased risk of the presence and severity of DPN, and AAR may be a novel and reliable marker for identifying subjects at high risk for DPN in patients with T2DM, however, the underlying mechanisms potentially responsible for the association remain unclear.

Growing evidence suggests that NAFLD is closely associated with the presence of DPN (50–52), while IR has been suggested to play a central role in the development and progression of NAFLD (53). In the present study, we found that patients with DPN had significantly lower HSL, which is a accurate proxy of NAFLD that can assess liver steatosis in predominantly Asian populations (30), and prevalence of NAFLD than those without DPN. Moreover, the logistic regression analysis revealed that HSL, TyG, a biochemical marker of IR (28), and prevalence of NAFLD were significantly associated with the presence of DPN. Our findings are largely in line with results from prior studies (50, 51, 54, 55). Yan et al. reported that patients with NAFLD diagnosed earlier than T2DM had a lower prevalence of DPN compared with those with T2DM diagnosed earlier than NAFLD or those with T2DM only (51). Another cross-sectional study demonstrated that the prevalence of NAFLD in Chinese T2DM patients with DPN was significantly lower than those without DPN, and NAFLD was negatively correlated with the prevalence of DPN (50). Recently, Zhao and colleagues revealed that a higher level of AUC of C-peptide was inversely associated with prevalence of diabetic neuropathy, and positively associated with homeostasis model assessment of IR index and NAFLD in 885 patients with T2DM (54). Similar results were also obtained by Guo et al. in T2DM patients (55). Together, these lines of evidence, combined with our results, suggest that NAFLD and its key component IR may protect against the development and progression of DPN in T2DM patients. Moreover, we demonstrated that patients with higher AAR quartiles tended to have longer diabetic duration and lower TyG, HSL, and prevalence of NAFLD compared to those with lower quartiles. Additionally, the Spearman correlation analysis revealed that AAR was negatively associated with HSL, TyG, and prevalence of NAFLD. Qiao et al. found that C-peptide and insulin levels progressively decreased (inadequate insulin secretion) and IR was relatively low because of weakened or even deterioration of pancreatic islet β cell function induced by long-term hyperglycemia along with increased diabetic duration, leading to increased prevalence of DPN (56). Combined, these data suggest that there might be a negative correlation between AAR and IR and NAFLD, and higher AAR might contribute to the development of DPN through a complex mechanism associated with IR and NAFLD; however, the mechanism of action needs to be further investigated.

Numerous studies have demonstrated that low-grade inflammation and oxidative stress are also contributing factors in the development and progression of DPN (7, 21, 49). Serum ALB is the most abundant circulating protein in blood synthesized and secreted from liver cells. It has been reported that serum ALB is the major source of extracellular reduced sulfhydryl groups, which act as potent scavengers of reactive oxygen and nitrogen species, thus constituting the dominant antioxidant in the circulatory system (57, 58). In addition, some substances such as nitric oxide and bilirubin are carried by serum albumin and provide additional protection against oxidative stress (57). Also, serum ALB can bind various inflammatory mediators and inhibit the secretion of pro-inflammatory cytokines, thus involving in regulating the inflammatory immune response and endothelial stabilization (58, 59). It has been suggested that elderly people are susceptible to oxidative stress due to a decline in the inefficiency of their endogenous antioxidant systems (60), and oxidative stress and inflammatory mediators increase with aging (61). In the present study, we found that patients with DPN had significantly older age and lower serum ALB than those without DPN. The logistic regression analysis revealed that age and serum ALB were significantly and independently associated with the presence of DPN after multivariate adjustment. Our findings were in agreement with previous studies (62–65) showing that serum ALB has neuroprotective effects *via* its antioxidant/anti-inflammatory activity, and its lower level was related to abnormal peripheral nerve function and a significantly increased risk of DPN, and older age is a risk factor for DPN, providing further evidence that inflammation and oxidative stress induced by lower serum ALB and older age may be closely associated with the presence of DPN. Moreover, patients with higher AAR quartiles tended to be relatively older and had significantly lower serum ALB compared to those with lower quartiles. Additionally, AAR was positively associated with age, and negatively with serum ALB. Our findings were in agreement with most previous studies (15, 66–69). Liu et al. reported that Chinese hypertensive patients with higher AAR had significantly lower levels of serum ALB and other endogenous antioxidant substances compared with those with lower AAR (15). Evidence from an animal study has also suggested that mice with elevated AAR had a reduced ability to carry oxygen, which was accompanied by significantly elevated levels of markers of oxidative stress (66). Several studies also have announced that hepatic steatosis assessed by AAR is associated with increased production of interleukin-6 and other pro-inflammatory cytokines by hepatozytes and nonperynchymal cells (67–69). Together, these lines of evidence, combined with our results, suggest that higher AAR may be closely associated with increased inflammation and oxidative stress, and inflammation and oxidative stress induced by lower serum ALB and older age might at least partially mediate the potential relationship between AAR and DPN, but larger, well-characterized, prospective research is still needed to validate these findings.

Experimental and epidemiological studies have shown that atherosclerotic vascular disease plays a critical role in the development and progression of DPN (23, 64). In the present study, we found that patients with DPN had significantly higher prevalence of DFU and PAD, two major diabetic macrovascular complications associated with atherosclerosis, than those without DPN. Moreover, the logistic regression analysis revealed that the prevalence of PAD was significantly positively associated with the presence of DPN, while DFU was an independent risk factor for DPN. Our findings further provided evidence that atherosclerotic vascular disease, especially DFU, and DPN are closely interconnected, and nerve ischemia associated with vascular dysfunction may contribute to nerve damage, eventually leading to the development of DPN. Moreover, patients with higher AAR quartiles tended to have higher prevalence of DFU and PAD and lower ABI, and AAR was significantly positively associated with prevalence of PAD and DFU, which was in general agreement with **two** previous studies (15, 70). A cross-sectional study that included 10900 Chinese adults with hypertension discovered that a high AAR was independently and positively associated with associated with PAD risk (15). Similarly, another cross-sectional study conducted by Rief and his colleagues reported that an elevation in AAR is significantly associated with an increased risk of occurrence of critical limb ischemia, independently of well-established risk factors, in patients with peripheral arterial occlusive disease (70). Together, these results indicate that high AAR might be linked to PAD and critical limb ischemia, an important risk factor for DFU, and vascular damage, especially DFU, might be associated with the presence of DPN. It is well-known that AST is abundantly present in many different types of tissue in addition to the liver, such as skeletal, cardiac, smooth muscle, kidney, and brain, whereas ALT is low in cells other than hepatocytes (71). Thus, an increased vulnerability of the liver and several other tissue associated with AST distribution to ischemia due to vascular damage caused by DFU would lead to an higher AAR in T2DM patients with DPN (6, 71, 72). However, the exact mechanism responsible for the relationship between AAR and DPN is still obscure and required further investigation.

Some potential limitations of our study should be noted. First, the causality of the relationship between AAR and DPN could not be established because this design of the present study is cross-sectional. Second, individuals with T2DM are at high risk for both microvascular complications and macrovascular complications, and thus may usually needs to take multiple medications at the same time, of which might affect liver transaminase due to potential drug-drug interactions. However, detailed medication history, such as statins, for these subjects was unavailable. Third, the present study population consisted of T2DM inpatients of Chinese Han ancestry, who generally had more serious illness than diabetic outpatients, and thus, our findings cannot to be extrapolated to diabetic outpatients and other types of diabetes with different ethnic back grounds. Finally, it has been reported that a sedentary lifestyle and unhealthy dietary habits are associated with elevated liver enzyme levels (73), however, insufficient data were available for the information about their diet and lifestyle, which might have influenced the results. Despite these limitations, this study has several strengths such as a relatively large sample size, use of a standardized method at a single center, and thorough adjustment for possible confounding variables.

In conclusion, the present study demonstrated that AAR was significantly increased in T2DM patients with DPN, and was independently associated with increased risk of presence of DPN in Chinese patients with T2DM, thereby suggesting that AAR may serve as an useful and reliable biomarker of DPN, and highlighting that it is crucial to pay more attention to T2DM patients with high AAR to further prevent and reduce the development of DPN and related unfavorable health outcomes.

## Data availability statement

The original contributions presented in the study are included in the article/**Supplementary Material**. Further inquiries can be directed to the corresponding author.

## Ethics statement

The studies involving human participants were reviewed and approved by the human research ethics committee of the Affiliated Hospital of Southwest Medical University. The patients/participants provided their written informed consent to participate in this study. Written informed consent was obtained from the individual(s) for the publication of any potentially identifiable images or data included in this article.

## Author contributions

All the authors contributed significantly to the manuscript. PY conducted the population study, analysed and interpreted the data, and drafted the manuscript. QW significantly revised the draft, interpreted the data, and involved in data analyses. YW, XD, XW, and QT conducted the study, collected the information and participated in data interpretation. XC, YX, JZ, and YM involved in the sample test, data management and draft revision. QW is the PI of project, who designed the study and critically revised the manuscript. All authors read and approved the final manuscript. All authors contributed to the article and approved the submitted version.
