# Supplementary material for: Aspartate aminotransferase/alanine aminotransferase ratio was associated with type 2 diabetic peripheral neuropathy in a Chinese population: A cross-sectional study
Source: Front Endocrinol (Lausanne). 2023 Feb 27;14:1064125. doi: 10.3389/fendo.2023.1064125 (PMC9998996; doi:10.3389/fendo.2023.1064125)
Supplement: Supplementary file 1 [file Table_1.doc]

**Supplementary Table 1 AAR levels and other clinical characteristics in T2DM patients by DPN**

| **Variables** | **no DPN** | **DPN** | ***P*-value** |
| --- | --- | --- | --- |
|  | **(n =1326 )** | **(n = 236)** |  |
| Male (n, %) | 646 (48.72%) | 128 (54.24%) | 0.118 |
| Age (years) | 58.51±11.12 | 66.61±9.86 | 0.000 |
| BMI (kg/m^2^) | 24.31±3.61 | 23.55±3.87 | 0.003 |
| Diabetic duration (years) | 7.07±6.16 | 10.23±7.32 | 0.000 |
| Smoking (n, %) | 286 (21.57%) | 47 (19.92%) | 0.568 |
| SBP (mmHg) | 132.01±20.46 | 135.08±22.62 | 0.028 |
| DBP (mmHg) | 72.44±12.03 | 69.95±12.77 | 0.005 |
| TC (mmol/L) | 4.90±1.36 | 4.65±1.31 | 0.005 |
| TG (mmol/L) | 2.44±2.75 | 1.87±1.40 | 0.000 |
| HDL-C (mmol/L) | 1.17±0.37 | 1.18±0.36 | 0.818 |
| LDL-C (mmol/L) | 2.79±1.01 | 2.75±0.96 | 0.517 |
| ApoA (g/L) | 1.34±0.30 | 1.27±0.34 | 0.000 |
| ApoB (g/L) | 0.91±0.28 | 0.91±0.35 | 0.418 |
| TyG | 9.50±1.07 | 9.35±1.10 | 0.124 |
| AIP | 0.50±0.02 | 0.31±0.05 | 0.005 |
| AC | 3.59±2.56 | 3.13±1.30 | 0.022 |
| FBG (mmol/L) | 10.75±5.13 | 11.54±5.53 | 0.017 |
| HbA1c (%) | 9.42±2.47 | 10.10±2.62 | 0.000 |
| ALT (U/L) | 24.04±18.09 | 18.16±12.93 | 0.000 |
| AST (U/L) | 22.38±16.69 | 18.98±8.97 | 0.000 |
| AAR | 1.07±0.44 | 1.24±0.53 | 0.000 |
| TBIL (μmol/L) | 12.49±5.71 | 11.03±4.96 | 0.000 |
| GGT (U/L) | 44.67±2.88 | 38.54±4.29 | 0.009 |
| Serum albumin (g/L) | 41.54±4.73 | 37.84±4.72 | 0.000 |
| WBC (*10^9^ /L) | 6.70±2.31 | 7.40±2.85 | 0.000 |
| Neutrophil (*10^9^ /L) | 4.46±2.16 | 5.35±2.70 | 0.000 |
| Lymphocyte (*10^9^ /L) | 1.68±0.63 | 1.44±0.56 | 0.000 |
| NLR | 3.14±2.72 | 4.54±4.21 | 0.000 |
| RDW (%) | 13.15±1.28 | 13.26±1.27 | 0.256 |
| Fibrinogen (g/L) | 3.53±1.23 | 4.38±1.63 | 0.000 |
| Serum UA (μmol/L) | 314.73±108.53 | 326.46±107.36 | 0.059 |
| Serum Cr (μmol/L) | 70.95±45.01 | 89.39±56.69 | 0.000 |
| eGFR (mL/min/1.73 m^2^) | 94.41±25.05 | 77.92±27.64 | 0.000 |
| Urinary ACR (mg/g) | 208.15±22.92 | 408.06±67.52 | 0.000 |
| ABI | 1.04±0.13 | 0.94±0.24 | 0.000 |
| VPT (V) | 12.94±4.73 | 35.86±8.76 | 0.000 |
| HSL | 33.73±5.39 | 31.91±5.55 | 0.000 |
| Dyslipidemia (n, %) | 726 (54.75%) | 118 (50.00%) | 0.177 |
| NAFLD (n, %) | 626 (47.21%) | 87 (36.86%) | 0.003 |
| MetS (n, %) | 621 (46.83%) | 119 ( 50.42%) | 0.309 |
| DN (n, %) | 507 (38.24%) | 150 (63.56%) | 0.000 |
| DR (n, %) | 151 (11.39%) | 50 (21.19%) | 0.000 |
| Hypertension (n, %) | 679 (51.21 %) | 149 ( 63.14%) | 0.001 |
| CHD (n, %) | 99 (7.47%) | 41 (17.37%) | 0.000 |
| DFU (n, %) | 61 (4.60%) | 54 (22.88%) | 0.000 |
| PAD (n, %) | 90 (6.79%) | 64 (27.12%) | 0.000 |

Data are mean ±SD. SD, standard deviation; DPN, diabetic peripheral neuropathy; BMI, body mass index; SBP, systolic blood pressure; DBP, diastolic blood pressure; TC, total cholesterol; TG, triglyceride; HDL-C, high-density lipoprotein cholesterol; LDL-C, low-density lipoprotein cholesterol; apoA, apolipoprotein A; apoB, apolipoprotein B; TyG, triglyceride-glucose; AIP, atherogenic index of plasma; AC, atherogenic coefficient; FBG, fasting blood glucose; HbA1c, glycated hemoglobin A1c; ALT, alanine aminotransferase; AST, aspartate aminotransferase; AAR, aminotransferase to alanine aminotransferase ratio; TBIL, total bilirubin; GGT, gamma-glutamyl transferase; WBC, white blood cell; NLR, neutrophil to lymphocyte ratio; RDW, red blood cell distribution width; UA, uric acid; Cr, creatinine; eGFR, estimated glomerular filtration rate; ACR, albumin- to-creatinine ratio; ABI, ankle-brachial index; VPT, vibration perception threshold; HSL, hepatic steatosis index; NAFLD, nonalcoholic fatty liver disease; MetS, metabolic syndrome; DN: diabetic nephropathy; DR: diabetic retinopathy; CHD: coronary heart disease; DFU: diabetic foot ulceration; PAD: peripheral arterial disease.
